# Using Biometric Sensor Data to Monitor Cancer Patients During Radiotherapy: Protocol for the OncoWatch Feasibility Study

**DOI:** 10.2196/26096

**Published:** 2021-05-13

**Authors:** Cecilie Holländer-Mieritz, Ivan R Vogelius, Claus A Kristensen, Allan Green, Judith L Rindum, Helle Pappot

**Affiliations:** 1 Department of Oncology, Rigshospitalet University of Copenhagen Copenhagen Denmark; 2 Telemedical Knowledge Center Capital Region of Denmark Hillerød Denmark

**Keywords:** biometric sensor technology, cancer, head and neck cancer, home monitoring, patient-generated health data, radiotherapy, sensor, smartwatch

## Abstract

**Background:**

Patients with head and neck cancer (HNC) experience severe side effects during radiotherapy (RT). Ongoing technological advances in wearable biometric sensors allow for the collection of objective data (eg, physical activity and heart rate), which might, in the future, help detect and counter side effects before they become severe. A smartwatch such as the Apple Watch allows for objective data monitoring outside the hospital with minimal effort from the patient. To determine whether such tools can be implemented in the oncological setting, feasibility studies are needed.

**Objective:**

This protocol describes the design of the OncoWatch 1.0 feasibility study that assesses the adherence of patients with HNC to an Apple Watch during RT.

**Methods:**

A prospective, single-cohort trial will be conducted at the Department of Oncology, Rigshospitalet (Copenhagen, Denmark). Patients aged ≥18 years intended for primary or postoperative curatively intended RT for HNC will be recruited. Consenting patients will be asked to wear an Apple Watch on the wrist during and until 2 weeks after RT. The study will include 10 patients. Data on adherence, data acquisition, and biometric data will be collected. Demographic data, objective toxicity scores, and hospitalizations will be documented.

**Results:**

The primary outcome is to determine if it is feasible for the patients to wear a smartwatch continuously (minimum 12 hours/day) during RT. Furthermore, we will explore how the heart rate and physical activity change over the treatment course.

**Conclusions:**

The study will assess the feasibility of using the Apple Watch for home monitoring of patients with HNC. Our findings may provide novel insights into the patient’s activity levels and variations in heart rate during the treatment course. The knowledge obtained from this study will be essential for further investigating how biometric data can be used as part of symptom monitoring for patients with HNC.

**Trial Registration:**

ClinicalTrials.gov NCT04613232; https://clinicaltrials.gov/ct2/show/NCT04613232

**International Registered Report Identifier (IRRID):**

PRR1-10.2196/26096

## Introduction

A promising type of patient-generated health data is biometric sensor data [1]. A “wearable” is a device that uses biometric sensors to monitor variables such as heart rate, blood glucose, and skin temperature [2-4]. One type of consumer wearables are smartwatches, which are also commonly used as fitness trackers [5].

In the oncological setting, most patients receive their treatment at outpatient clinics, which implies that they spend most of their time outside the hospital. The patients are instructed to contact the oncology department if they experience side effects or other alerting symptoms. If their symptoms deteriorate, they might need acute hospital admission. In the hospital, the patients are monitored with vital signs depending on their condition. Changes in vital signs can alert the health care professionals and can contribute to optimizing supportive treatment. In the outpatient clinics, the patients are seen at planned intervals. During regular consultations, side effects are discussed with the patient, and the patient’s health status is evaluated.

The symptoms and side effects experienced by the patient during cancer treatment depend on the cancer type, treatment type, and comorbidity. Patients with head and neck cancer (HNC) experience severe acute side effects during radiotherapy (RT), including pain, dysphagia, and dehydration [6-8]. At the annual American Society of Clinical Oncology meeting in 2018, Peterson et al [9,10] presented the findings of their randomized clinical trial, which showed that patients undergoing RT for HNC experienced less severe symptoms if they sent their daily monitored weight and blood pressure to their clinician.

Systematic home monitoring with a sensor device may have the potential to detect symptoms such as dehydration and increased pain [11], and biometric sensors in consumer wearables such as a smartwatch have made it possible to obtain objective measures with minimal burden to the patient [12]. However, studies on wearable sensor devices for home monitoring in the health care setting are limited [13-15]. We have previously shown that in oncological clinical trials, wearable sensors have primarily been used to monitor physical activity and the circadian rhythm [16]. The most frequent cancer types in these studies were breast cancer, followed by gastrointestinal and lung cancer. Few studies focused on biometric sensor data as a supplement to symptom monitoring [12,17-19]. Knowledge about the adherence to the wearable is important when introducing new technologies, and this is generally a lesser known area [16]. It remains unknown whether patients with a moderate-to-severe symptom burden, such as those with HNC undergoing RT, can adhere to the use of a wearable during treatment.

Here we describe the design of the OncoWatch 1.0 feasibility study. This study is aimed to determine the adherence to using an Apple Watch during curatively intended RT for HNC. Our findings may provide novel insights into the patients’ activity levels and variations in the heart rate during their treatment course.

## Methods

### Patients and Recruitment

Ten patients from Denmark, who are aged ≥18 years and are intended for primary or postoperative curative RT (5-6 fractions/week/up to 34 fractions) for squamous cell carcinoma of the head and neck at the Department of Oncology, Rigshospitalet (Copenhagen, Denmark) will be enrolled. Other inclusion criteria are the ability to read and speak Danish, having no serious cognitive deficits, and providing written informed consent to participate in the study. Patients will be included consecutively at their initial visit at the Department of Oncology, Rigshospitalet.

### Design

**Figure 1:**
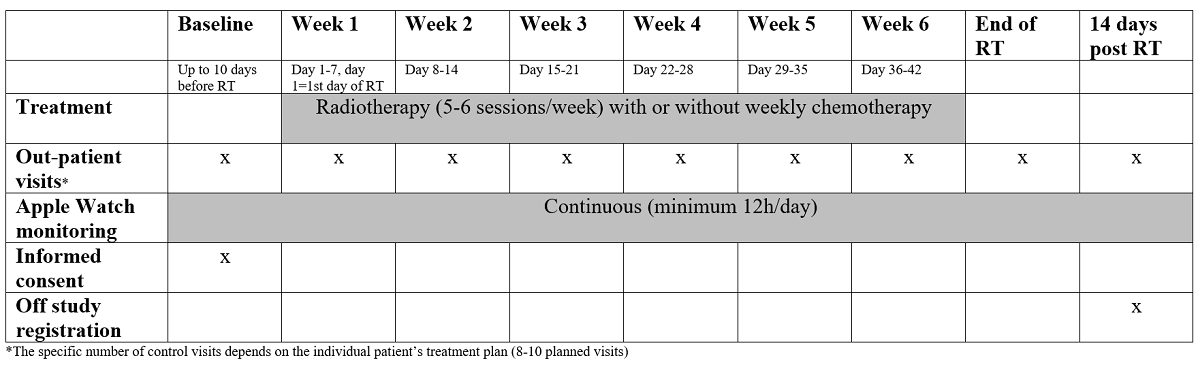
Overview of the OncoWatch feasibility study assessment times, tasks, and measures. RT: radiotherapy.

The trial is an explorative feasibility study investigating the adherence to the Apple Watch, changes in the heart rate and physical activity during RT for HNC. This trial has been registered on ClinicalTrials.gov (protocol# NCT04613232). The study will be performed in a public health care system, which implies that the patients will have access to the hospital free of charge. The research intervention is continuous monitoring of heart rate and physical activity (minimum 12 hours/day) with a smartwatch connected to a smartphone (Figure 1). The patients will be asked to wear the smartwatch from baseline until 14 days after the end of treatment. Patients will go off study when they have attended their control visit 2 weeks after the end of RT or if they no longer want to participate. Inclusion in the study will have no interference with their oncological treatment. The primary investigator will oversee that data are regularly transmitted to the database.

The study will follow the General Data Protection Regulation and is registered at the capital region of Denmark (ID. P- P-2019-797). In a Danish setting, the study does not need approval from the National Committee on Health Research Ethics. This study has been approved by the local division for information technology and medical technology in the capital region of Denmark. This study is a collaboration between the Department of Oncology, Rigshospitalet, and the Telemedical Knowledge Center at the capital region of Denmark. The Telemedical Knowledge Center supports and improves the use of telemedicine in in the capital region of Denmark.

### Hardware

The wearable is an Apple Watch Series 5 worn on the wrist. The watch is connected to an iPhone 8 device.

The smartwatch and smartphone will be supplied by the hospital. The patients will return the devices at study termination. The patients will not be able to use their own device, neither phone nor smartwatch. Only the patient is allowed to wear the smartwatch during the study period. The watch and phone can only be operated with a unique password. The patients will be responsible for charging the watch and phone. The patients will not receive any rewards or financial support for participating in the study.

### Software

ZiteLab ApS has developed the OncoWatch app, which collects data from the Apple HealthKit and sends it to a secure cloud server [20]. The primary investigator has access to the database. In this feasibility study, the patients are not supposed to interact with the smartwatch or the OncoWatch app.

**Figure 2:**
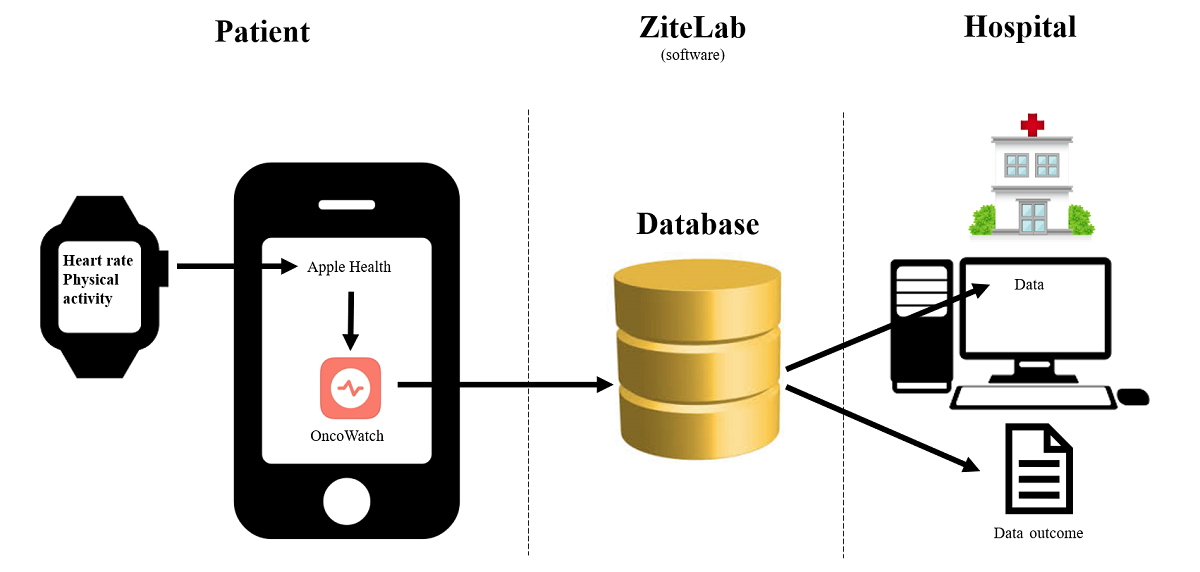
Framework for the OncoWatch 1.0 feasibility study.

The patients will be assigned an account before they can use the app (Figure 2).

### Statistical Analysis

#### Endpoints

To determine the feasibility and adherence to the smartwatch, the primary endpoint is number of patients who could wear the device minimum 12 hours/day during the study period (from baseline until 2 weeks after the end of RT). Secondary endpoints are the percentage of successful data acquisition events and variations in heart rate and physical activity. Heart rate at rest and the average heart rate during movement will be recorded. Physical activity, defined as steps per day, is registered.

Clinical data from all patients will be collected and will include age, stage, treatment regimen, information on hospital admissions, and toxicity score. The Danish Head and Neck cancer Study Group toxicity score is an objective grading of the patient’s symptoms assigned by the clinician [21], which is determined using a categorical scale of 0-4 with 0=“no or nothing” and 4=“severe.”

The statistical analysis will include descriptive statistics of the patients included in the study. Descriptive data will be collected and analyzed using SPSS Statistics (IBM Corp) or SAS (SAS Institute). Data will be captured in RedCap. Missing data will be described.

#### Power

This is a feasibility study and does not require a power calculation. The present study data will be used to calculate adequate sample sizes in future randomized clinical trials.

#### Ethical Considerations

The patients will receive verbal and written information and will be required to provide written informed consent, which they can withdraw at any time. The patient will be handed a phone and watch by the hospital.

## Results

Patient recruitment was initiated in March 2021.

## Discussion

This study will assess the feasibility of using the Apple Watch for home monitoring of patients with HNC. Our findings may provide novel insights into the patients’ activity levels and variations in the heart rate during their treatment course. The knowledge obtained from this study will be essential for further investigating how biometric data can be used as part of symptom monitoring for patients with HNC.

## Abbreviations

HNC: head and neck cancer
RT: radiotherapy
